# Chemerin is a novel biomarker of acute coronary syndrome but not of stable angina pectoris

**DOI:** 10.1186/s12933-014-0145-4

**Published:** 2014-11-01

**Authors:** Qingwei Ji, Yingzhong Lin, Zhishan Liang, Kunwu Yu, Yuyang Liu, Zhe Fang, Ling Liu, Ying Shi, Qiutang Zeng, Chao Chang, Meng Chai, Yujie Zhou

**Affiliations:** Department of Cardiology, Beijing Anzhen Hospital, Capital Medical University, Beijing Institute of Heart Lung and Blood Vessel Disease, The Key Laboratory of Remodeling-related Cardiovascular Disease, Ministry of Education, Beijing, 100029 China; Department of Cardiology, The People’s Hospital of Guangxi Zhuang Autonomous Region, Nanning, 530021 China; Institute of Cardiovascular Diseases, Union Hospital, Tongji Medical College, Huazhong University of Science and Technology, Wuhan, 430022 China

**Keywords:** Chemerin, Adiponectin, Acute coronary syndrome, Inflammation, Left ventricular function

## Abstract

**Background:**

Recent evidence demonstrated that the circulating adipokines were associated with the onset of acute coronary syndrome (ACS) including unstable angina pectoris (UAP) and acute myocardial infarction (AMI). As a novel adipokine, chemerin has been related to atherosclerosis and the presence of coronary artery disease. However, the plasma levels of chemerin in patients with ACS have yet to be investigated.

**Methods:**

Plasma levels of chemerin and adiponectin were measured by an enzyme-linked immunosorbent assay (ELISA) in 60 patients with stable angina pectoris (SAP), 60 patients with UAP, 60 patients with AMI and 40 control patients. Left ventricular end-diastolic diameter (LVEDD) and left ventricular ejection fraction (LVEF) were measured using a GE ViVid E7 ultrasonography machine, and the severity of coronary stenosis in patients was estimated with a Gensini coronary score following coronary angiography.

**Results:**

Plasma chemerin levels were significantly higher in ACS patients than in the control and SAP groups, while plasma adiponectin levels were significantly lower in ACS patients than the control group. A correlation analysis revealed that plasma chemerin levels were positively correlated with the levels of C-reactive protein (CRP) (r = 0.29, P < 0.01) and LVEDD (r = 0.27, P < 0.01) but negatively correlated with LVEF (r = -0.45, P < 0.01) and that plasma adiponectin levels were positively correlated with LVEF (r = 0.53, P < 0.01) but negatively correlated with CRP (r = -0.33, P < 0.01) and LVEDD (r = -0.30, P < 0.01). Although significant correlations between chemerin, adiponectin and BMI or the Gensini coronary score were found in patients with SAP, neither chemerin nor adiponectin was correlated with BMI and the Gensini coronary score in patients with ACS. Furthermore, both chemerin (OR 1.103, 95% CI 1.065 to 1.142; P = 0.001) and adiponectin (OR 0.871, 95% CI 0.776 to 0.970; P = 0.018) were independently associated with the presence of ACS.

**Conclusions:**

Chemerin is a novel biomarker of acute coronary syndrome but not of stable angina pectoris.

## Background

Coronary artery disease (CAD) is typically divided into different clinical types including stable angina pectoris (SAP) and acute coronary syndrome (ACS). ACS including unstable angina pectoris (UAP) and acute myocardial infarction (AMI) is the clinical definition of the critical phase of CAD which results primarily from a disruption of a coronary atherosclerotic plaque associated with partial or complete thrombotic vessel occlusion [1-4]. In contrast, SAP is the initial manifestation of ischemic heart disease in one half of patients and becomes a recurrent symptom in survivors of ACS that is generally due to one or more significant obstructive but more stable lesions in coronary arteries [1-4]. Although chronic inflammation is a characteristic shared between ACS and SAP, the inflammatory response is significantly more robust in ACS than in SAP [1-4]. In addition, the prognosis for ACS is poorer than for SAP, and revascularization therapy has to be performed promptly in ACS but deliberately in those SAP patients who had symptoms after optimal medial therapy or were performed by appropriate intracoronary assessment of hemodynamic relevance. Therefore, it is necessary and helpful to seek biomarkers associated with the presence of ACS.

Adipose tissue serves not only as a mass of fat for storing energy but also as an active endocrine organ that secretes various bioactive adipokines. Most adipokines, such as tumor necrosis factor (TNF)-α, interleukin (IL)-6, visfatin and leptin are well-known pro-inflammatory cytokines that accelerate atherosclerosis in experimental model [5-10], and contribute to the presence of ACS [11-14]. Other members of this group, such as adiponectin, are anti-inflammatory cytokines that play a protective role in atherosclerosis [15], and the relationships between the blood concentrations of this molecule and ACS have been examined extensively [16-21]. The results from our and others studies showed circulating adiponectin concentrations were significantly decreased in ACS compared to the control and SAP groups, suggesting that adiponectin is a biomarker of ACS [16-22]. In addition, some studies demonstrated that circulating adiponectin is significantly associated with the onset of coronary artery disease and myocardial infarction in subjects below the age of 60 [23,24], and all cause/cardio-vascular disease (CVD) mortality [25].

Chemerin, which is also known as tazarotene-induced gene 2 protein (TIG2) or retinoid acid receptor responder 2 (RARRES2), is a novel adipokine that plays a pivotal role in adipose differentiation, maturation and metabolism, regulation of immune response and insulin resistance [26-29]. In addition, chemerin acts as an inflammatory mediator to promote the migration of macrophages and immature dendritic cells and the production of pro-inflammatory cytokines. However, chemerin was also demonstrated to play an anti-inflammatory role as chemerin significantly prevented TNF-a-induced VCAM-1 expression and monocyte adhesion by inhibiting the activation of NF-КB and p38 *in vitro* [30]. Accumulating evidence demonstrates that high concentrations of circulating chemerin are associated with the presence of CAD, suggesting that circulating chemerin is a novel biomarker of CAD [31-34]. However, whether circulating chemerin levels are different in patients with SAP and patients with ACS has yet to be investigated.

In the present study, plasma levels of chemerin and adiponectin were measured in enrolled patients with SAP and ACS and the relationships between plasma levels of these two adipokines and other clinical parameters including were analyzed. Because previous studies showed that medication use such as statin regulates the secretion of adipokines [17], we also investigate the effects of medication use on plasma levels of chemerin and adiponectin.

## Methods

### Patients

We recruited 220 patients who underwent diagnostic coronary angiography between Augest 2012 and March 2013 in Beijing Anzhen Hospital, the People’s Hospital of Guangxi Zhuang Autonomous Region, and Union Hospital, China. The patients were classified into 4 groups: (1) Stable angina pectoris (SAP). The inclusion criteria for this group were typical exertional chest discomfort that was associated with down sloping or horizontal ST-segment depression >1 mm in an exercise test); (2) Unstable angina pectoris (UAP). The inclusion criteria for this group were chest pain at rest with definite ischemic electrocardiographic changes: ST-segment changes and/or T-wave inversions; (3) Acute myocardial infarction (AMI). The inclusion criteria for this group were myocardial infarction that was confirmed by a significant increase of troponin I and Creatine Kinase MB levels; and (4) Control group, which consisted of 40 subjects with normal coronary artery findings.

Written informed consent was obtained from each patient. The study was approved by the Ethics Committee of Beijing Anzhen Hospital, the People’s Hospital of Guangxi Zhuang Autonomous Region, and Union Hospital. Patients with valvular heart disease, thromboembolism, collagen disease, disseminated intravascular coagulation, advanced liver disease, renal failure, malignant disease, septicemia, other inflammatory disease, or current steroid therapy were excluded from the study.

### Clinical data collection

Clinical data were obtained upon admission to hospital. Demographic data, height, body weight, medical history, and medication use were recorded. Body mass index (BMI) was calculated as weight (kg) divided by square of height (m^2^).

### Blood samples measurements

In the AMI group, blood samples were obtained from the patients upon arrival at the emergency unit. Fasting blood samples were obtained the morning after admission for the rest of the study groups. The samples were collected in sodium heparin Vacutainers (Becton-Dickinson). Blood was centrifuged for 15 min at 3,000 × g and the plasma was stored at -80°C until further use.

The levels of plasma chemerin and adiponectin (R&D Systems, USA) were measured by an enzyme-linked immunosorbent assay (ELISA), following the manufacturer’s instructions. The ELISA intra-assay and inter-assay coefficients of variation were <5% and <10%, respectively. All of the samples were measured in duplicate.

The levels of lipid and lipoprotein fractions, fasting glucose, creatinine and C-reactive protein (CRP) at baseline were measured in central laboratory of Beijing Anzhen Hospital.

### Doppler echocardiography

The patients underwent M-mode and 2D-echocardiography using a GE ViVid E7 ultrasonography machine (GE Healthcare, America) with a transthoracic 1.5–4.3 MHz probe (M5S-D). Left ventricular end-diastolic diameter (LVEDD) and fractional shortening were measured. Left ventricular ejection fraction (LVEF) was calculated from apical four chambers position by the area–length method.

### Gensini score

The severity of coronary stenosis in patients was estimated with a Gensini coronary score following coronary angiography [35].

### Statistical analysis

All of the data were given as the mean ± SD. When comparing only 2 groups, Student’s *T*-test was used. For comparisons involving 3 or more groups, one-way ANOVA followed by Neuman-Keuls post-hoc test was used. Spearman’s correlation was used to calculate the correlations between the plasma chemerin levels and the other measured parameters. To identify the independent predictors of the presence of ACS, simple linear regression analyses were firstly performed and the candidate variables entered in the model included age, sex, BMI, hypertension, diabetes, smoking, lipid and lipoprotein fractions, fasting glucose, creatinine, CRP, chemerin and adiponectin, and then those variables exhibited a trend (p < 0.05) toward an association with the presence of ACS were entered in binary logistic regression analyses. Odds ratios (ORs) and 95% confidence intervals (CIs) were calculated. In all of the tests, a value of P < 0.05 was considered to be statistically significant.

## Results

### Clinical characteristics of the study population

The baseline characteristics of the four groups were shown in Table 1. In the SAP group (n = 60), 44 were men, and mean age was 61.9 ± 9.9 years. In the UAP group (n = 60), 37 were men, and mean age was 60.6 ± 9.9 years. In the AMI group (n = 60), 41 were men, and mean age was 62.9 ± 10.3 years. In the control group (n = 40), 29 were men, and mean age was 62.6 ± 7.9 years.

**Table 1:** **Clinical characteristics of patients**

**Characteristics**	**Control (n = 40)**	**SAP (n = 60)**	**UAP (n = 60)**	**AMI (n = 60)**
Age (years)	62.6 ± 7.9	61.9 ± 9.9	60.6 ± 9.9	62.9 ± 10.3
Sex (male/female)	29/11	44/16	37/23	41/19
Hypertension, n (%)	22 (55)	34 (58)	38 (61.3)	32 (61.2)
Diabetes, n (%)	6 (15)	20 (28)	14 (38.7)	20 (37.3)
Smoking, n (%)	14 (35)	26 (40)	23 (48)	28 (35.8)
BMI (Kg/m^2^)	23. 2 ± 3.9	24.8 ± 3.0*	25.8 ± 2.7*	25.9 ± 2.6*
TC (mmol/L)	4.52 ± 0.99	4.09 ± 0.95	4.53 ± 1.19	4.40 ± 0.86
TG (mmol/L)	1.68 ± 1.75	1.84 ± 1.04	1.94 ± 1.61	1.82 ± 1.42
LDL-C (mmol/L)	2.71 ± 0.87	2.38 ± 0.81	2.69 ± 1.16	2.71 ± 0.78
HDL-C (mmol/L)	1.20 ± 0.34	1.11 ± 0.41	1.09 ± 0.37	0.94 ± 0.26*
GLU (mmol/L)	5.07 ± 0.9	5.67 ± 2.03	6.22 ± 2.67	6.78 ± 2.91*
Creatinine (μmol/L)	76.28 ± 14.97	88.24 ± 23.03*	82.51 ± 20.29*	87.77 ± 35.23*
CRP (mg/L)	1.49 ± 0.99	2.61 ± 4.04	4.14 ± 4.09*	7.53 ± 7.08*
LVEF (%)	65.7 ± 5.0	62.6 ± 8.5	56.9 ± 12.5*	48.5 ± 10.4*
LVEDD (mm)	46.7 ± 3.2	48.1 ± 3.7	51.5 ± 7.6*	52.7 ± 5.5*
Gensini score	0	44.0 ± 30.2*	52.0 ± 28.4*	61.9 ± 34.5*
Medications, n (%)				
Aspirin	6 (15)	24 (40)	23 (38.3)	25 (41.7)
β-blocker	6 (15)	22 (36.7)	26 (43.3)	30 (50)
ACEI/ARB	10 (25)	22 (36.7)	26 (43.3)	21 (35)
CCB	16 (40)	32 (53.3)	28 (46.7)	26 (43.3)
Statin	9 (22.5)	22 (52)	21 (35)	20 (33.3)
Nitrate	9 (22.5)	9 (15)	16 (26.7)	17 (28.3)

The data are given as the mean ± SD or number of patients. SAP: stable angina pectoris; UAP: unstable angina pectoris; AMI: acute myocardial infarction; TC: total cholesterol; TG: total triglycerides; LDL-C: low-density lipoprotein cholesterol; HDL-C: high-density lipoprotein cholesterol; GLU: fasting glucose; LVEF: left ventricular ejection fraction; LVEDD: left ventricular end-diastolic dimension; ACEI: angiotensin-converting enzyme inhibitor; ARB: angiotensin receptor blocker; CCB: Calcium channel blocker.

*P < 0.05 vs. control.

No significant differences in age, sex, history of hypertension, diabetes or smoking were observed among the four groups. BMI, creatinine, CRP, LVEDD, and the Gensini score were significantly higher in the UAP and AMI groups than in the control group; in contrast, the LVEF in the UAP and AMI groups was lower than that of the control group (Table 1).

### Plasma adipokines analysis

**Figure 1:**
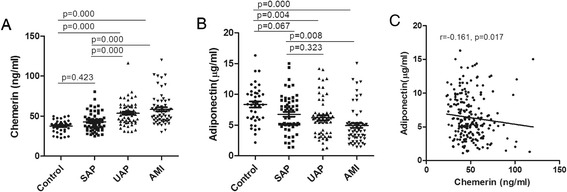
**The plasma adipokine concentrations in each group. A**: The plasma chemerin levels in patients with AMI and UAP were significantly increased compared to those of the control and SAP groups. **B**: The plasma adiponectin levels in patients with AMI and UAP were significantly reduced compared to those of the control group. **C**: The levels of chemerin were negatively correlated with the levels of adiponectin (r = 0.161, P = 0.017).

As shown in Table 2 and Figure 1, the plasma chemerin levels in patients with CAD, specifically patients in the AMI and UAP groups but not patients in the SAP group, were significantly increased compared to those of the control group. In addition, the plasma chemerin levels in the AMI and UAP groups were significantly increased compared to those in the SAP group. Further, the plasma adiponectin levels in patients with CAD, including the AMI and UAP groups, were significantly reduced compared to those in the control group. In addition, the plasma adiponectin levels in the AMI group but not the UAP group were significantly decreased compared to those in the SAP group (Figure 1). A correlation analysis demonstrated that plasma chemerin levels were weakly negatively correlated with plasma adiponectin levels (r = -0.16, P = 0.011) (Figure 1).

**Table 2:** **Plasma levels of chemerin and adiponectin in each groups and traditional risk factors**

	**No.**	**Chemerin (ng/ml)**	**Adiponectin (μg/ml)**
Control	40	37.55 ± 7.42	8.34 ± 3.06
CAD	180	51.95 ± 18.13**	5.95 ± 3.13**
SAP	60	42.85 ± 11.60	6.74 ± 3.01
UAP	60	53.62 ± 14.65**^,##^	6.19 ± 3.14**
AMI	60	59.38 ± 22.46**^,##^	4.92 ± 3.02**^,##^
Male	151	47.99 ± 18.12	6.29 ± 3.09
Female	69	52.26 ± 16.10	6.59 ± 3.59
Hypertension	126	50.10 ± 16.19	6.61 ± 3.12
Normotension	94	48.30 ± 16.19	6.08 ± 3.41
Diabetes	60	50.89 ± 22.02	6.54 ± 3.25
Non-diabetes	160	48.75 ± 15.65	6.33 ± 3.26
Smoking	91	47.24 ± 16.43	5.79 ± 2.97
Non-smoking	129	50.81 ± 18.28	6.80 ± 3.38

Note: The data are given as the mean ± SD. **P < 0.01 vs. Control, ^##^P < 0.01 vs. SAP.

We analyzed whether the levels of chemerin and adiponectin changed with traditional risk factors, such as sex, hypertension, diabetes, and smoking. As shown in Table 2, the circulating chemerin and adiponectin levels exhibited no significant differences in association with these traditional risk factors among all of the participants.

In addition, the plasma chemerin and adiponectin levels of all participants were summarized according to medication in the present study. The results demonstrated that the administration of aspirin, β-blocker, angiotensin-converting enzyme inhibitor (ACEI) or angiotensin receptor blocker (ARB), calcium channel blocker (CCB), statin and nitrate had no significant effects on plasma levels of chemerin and adiponectin (Table 3).

**Table 3:** **Plasma levels of chemerin and adiponectin in all patients according to medication**

**Medication**		**No.**	**Chemerin (ng/ml)**	**Adiponectin (μg/ml)**
Aspirin	Yes	78	51.15 ± 18.26	5.82 ± 3.04
	No	142	48.28 ± 17.18	6.69 ± 3.33
β-blocker	Yes	84	50.34 ± 16.80	5.94 ± 2.74
	No	136	48.71 ± 18.09	6.65 ± 3.51
ACEI/ARB	Yes	79	48.79 ± 15.95	6.01 ± 3.08
	No	141	49.63 ± 18.49	6.59 ± 3.32
CCB	Yes	103	49.72 ± 16.77	6.57 ± 3.34
	No	117	48.99 ± 18.34	6.22 ± 3.17
Statin	Yes	72	48.93 ± 19.48	6.63 ± 3.74
	No	148	49.53 ± 16.66	6.27 ± 2.99
Nitrate	Yes	51	48.59 ± 13.17	6.40 ± 2.89
	No	169	49.56 ± 18.75	6.38 ± 3.36

Note: The data are given as the mean ± SD.

### Relationship between plasma adipokine concentrations and the presence of ACS

**Figure 2:**
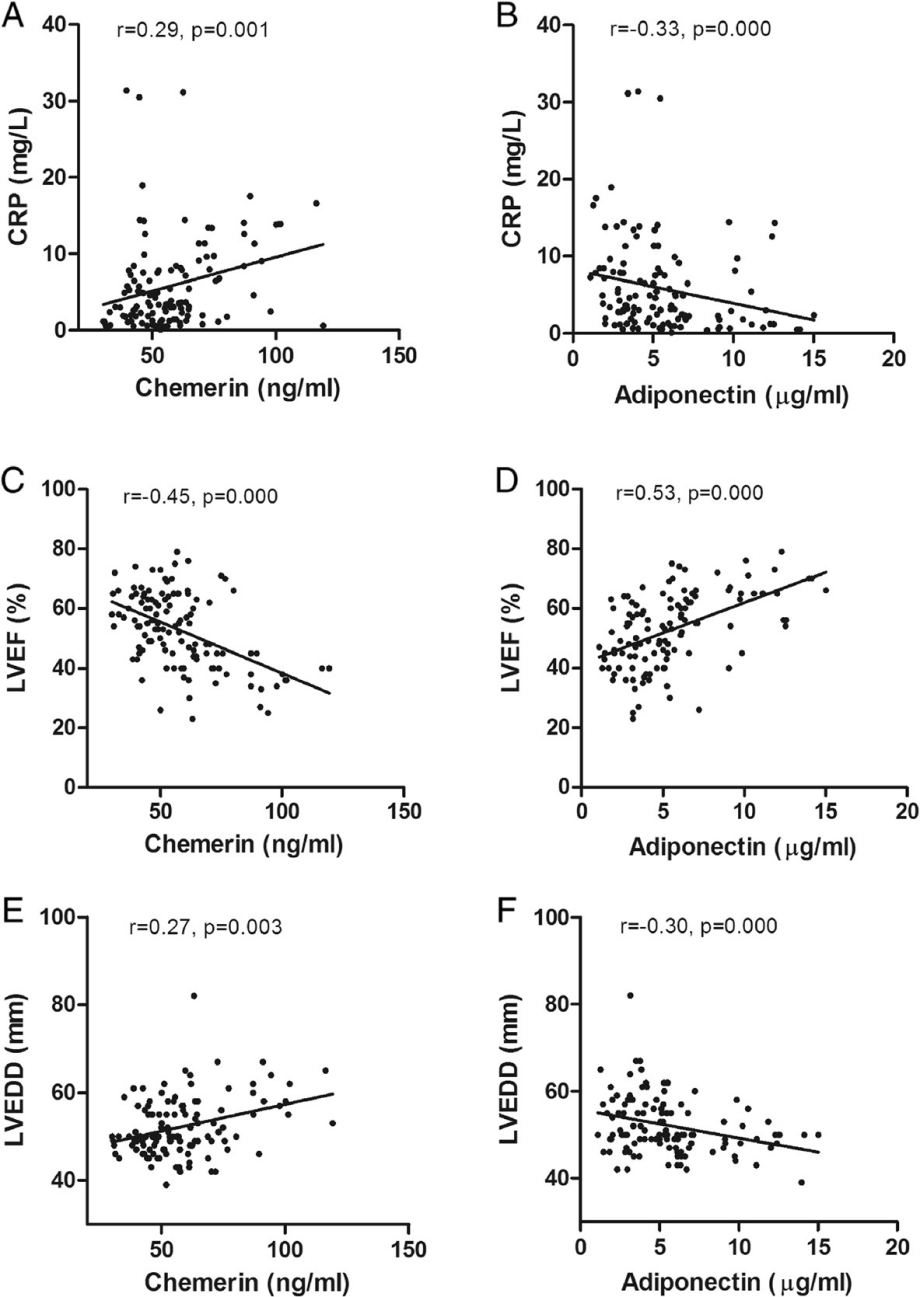
**Correlations between chemerin, adiponectin and other measured parameters in ACS. A**: Plasma levels of chemerin were positively correlated with CRP in patients with ACS. **B**: Plasma levels of adiponectin were negatively correlated with CRP in patients with ACS. **C**: Plasma levels of chemerin were negatively correlated with LVEF in patients with ACS. **D**: Plasma levels of adiponectin were positively correlated with LVEF in patients with ACS. **E**: Plasma levels of chemerin were positively correlated with LVEDD in patients with ACS. **F**: Plasma levels of adiponectin were negatively correlated with LVEDD in patients with ACS.

Because plasma levels of chemerin and adiponectin were significantly regulated in patients with ACS, we analyzed the correlation between the plasma chemerin and adiponectin levels and clinical parameters in ACS. The results showed that plasma levels of chemerin were positively correlated with CRP and LVEDD but negatively correlated with the LVEF, and the plasma levels of adiponectin were positively correlated with the LVEF but negatively correlated with TC, LDL-C, CRP and LVEDD (Table 4 and Figure 2). Although significant correlations between chemerin (r = 0319, P = 0.013), adiponectin (r = -0.262, P = 0.043) and BMI were found in patients with SAP, neither chemerin (r = 0.164, P = 0.075) nor adiponectin (r = -0.100, P = 0.275) was correlated with BMI in patients with ACS. In addition, although significant correlations between chemerin (r = 0.279, P = 0.031), adiponectin (r = -0.486, P = 0.000) and the Gensini score were found in SAP, neither chemerin (r = 0.098, P = 0.286) nor adiponectin (r = -0.074, P = 0.415) was correlated with the Gensini coronary score in ACS. However, there were no correlations between the levels of chemerin and adiponectin and the other parameters including age, TG, HDL-C, fasting glucose and creatinine.

**Table 4:** **Correlations between chemerin, adiponectin and other measured parameters in ACS**

**Characteristics**	**Chemerin (ng/ml)**	**Adiponectin (μg/ml)**
Age (years)	0.11	-0.07
BMI (Kg/m^2^)	0.16	-0.10
TC (mmol/L)	0.10	-0.25**
TG (mmol/L)	0.09	0.11
LDL-C (mmol/L)	0.10	-0.32**
HDL-C (mmol/L)	0.05	0.02
GLU (mmol/L)	0.15	-0.10
Creatinine (μmol/L)	0.11	-0.13
CRP (mg/L)	0.29**	-0.33**
LVEF (%)	-0.45**	0.53**
LVEDD (mm)	0.27**	-0.30**
Gensini score	0.10	-0.07

**P < 0.01.

### Simple linear regression and binary logistic regression analyses

To determine the independent predictors of the presence of ACS, we performed simple linear regression and binary logistic regression analyses. Simple logistic regression analysis demonstrated that fasting glucose, TC, HDL-C, LDL-C, CRP, adiponectin, and chemerin exhibited a trend (P < 0.05) toward an association with the presence of ACS. A binary logistic regression analyses was then performed using a model in which variables were included fasting glucose, TC, HDL-C, LDL-C, CRP, adiponectin, and chemerin. The results demonstrated that glucose (OR 1.288, 95% CI 1.064 to 1.558; p = 0.009), CRP (OR 1.205, 95% CI 1.070 to 1.357; P = 0.002), adiponectin (OR 0.871, 95% CI 0.776 to 0.970; P = 0.018), and chemerin (OR 1.103, 95% CI 1.065 to 1.142; P = 0.001) were independently associated with the presence of ACS.

## Discussion

Similar to our previous study [17], the present results demonstrated that plasma levels of adiponectin were significantly reduced in ACS patients compared to the control group, and that adiponectin was independently associated with the presence of ACS. Furthermore, the results also demonstrated that plasma levels of chemerin were significantly increased in patients with UAP and AMI compared to the control and SAP groups, and negatively correlated with plasma adiponectin levels. In addition, the binary logistic regression analyses demonstrated that a high level of chemerin was independently associated with the presence of ACS. Although a number of studies reported a high level of circulating chemerin in CAD [31-33], our present study found for the first time that plasma chemerin may be a novel biomarker of ACS but not of SAP, providing a novel insight into understand the complex relationship between chemerin and CAD. These results were consistent with another study in which there were no difference in chemerin levels between asymptomatic type 2 diabetic patients with CAD and without CAD, suggesting that chemerin is not a biomarker of patients with stable CAD [36].

Chemerin was initially identified from psoriatic skin lesions [26], and was subsequently proved to be a functional protein in human inflammatory fluids that served as the ligand of ChemR23 [27]. Both chemerin and ChemR23 are expressed at their highest levels in white adipose tissue, and the chemerin/ChemR23 axis plays a critical role in adipogenesis and adipocyte metabolism [28]. Beyond these observations, accumulating experimental evidence established a pleiotropic role for chemerin in diverse biological processes including the regulation of immune response, inflammation, insulin resistance and angiogenesis [29]. Data showed that chemerin promotes the migration of macrophage and immature dendritic cell [27]. It is well-known that a macrophage-to-foam cell switch elicits the initiation and development of atherosclerosis, and increased accumulation of macrophages induce the rupture of plaque and the thrombus formation in advanced atherosclerosis [1-4]. Therefore, chemerin may be involved in different stages of atherosclerosis through regulating the migration of macrophage. In contrary, accumulating evidence demonstrated that adiponectin not only suppresses foam cell transformation but also inhibits the pro-coagulant activity of macrophage [37]. In the present study, a high level of chemrin and a low level of adiponectin were detected in ACS, suggesting the imbalance of pro-inflammatory/anti-inflammatory adipokines may be associated with the presence of ACS by regulation the activity of macrophage. However, this is still a hypotheses which need be clarified in the future.

A large number of clinical studies demonstrated that elevated levels of circulating chemerin were associated with the onset of many traditional risk factors of CAD, such as sex [38], hypertension [39], and diabetes [40]. However, those findings were not observed in the present study. Data from previous studies revealed that medication use effectively regulates the secretion of chemerin and adiponectin [16,41]. More recently, a CAD case–control study found that plasma chemerin levels were significantly higher in CAD patients who were not treated with aspirin than in patients treated with aspirin and in control subjects [34]. Therefore, we analyzed the effect of medication on chemerin and adiponectin. The results revealed no difference in circulating levels of chemerin and adiponectin based on aspirin, β-blocker, ACEI or ARB, CCB, statin, or nitrate use.

Bioactive adipokines are secreted by adipose tissue and the list of adipokines has reached hundreds of factors. Accumulating evidence established that obesity, characterized by a condition of excess adipose tissue, resulted in the imbalance of pro-inflammatory/anti-inflammatory adipokines, and therefore deeply involved in the development of atherosclerotic disease [6,42,43]. The point that adipokine is the bridge between obesity and atherosclerosis is no longer controversial. In the present study, we found positive correlation between chemerin and BMI, and inverse correlation between adiponectin and BMI in patients with SAP, which was consistent with many previous studies [22,32,33]. However, neither chemerin nor adiponectin was correlated with BMI in patients with ACS, suggesting the regulation in adipokine production is more complicated in ACS.

Numerous studies demonstrated that circulating chemerin levels were correlated with lipid and lipoprotein fractions, fasting glucose, and various markers of inflammation [31-33,40]. Although no correlations between chemerin and lipid and lipoprotein fractions, fasting glucose, and creatinine were observed in the present study, the results demonstrated that CRP, which is an established marker of inflammation, was positively correlated with chemerin but negatively correlated with adiponectin, suggesting that inflammation may be the bridge between adipokines and ACS. Correlation analyses also demonstrated that increased chemerin levels were positively correlated with LVEDD but negatively correlated with LVEF and that reduced adiponectin levels were positively correlated with LVEF but negatively correlated with LVEDD in ACS. It is well-known that ACS patients with severe systemic inflammation and impaired cardiac function are at higher risk for death and future ischemic events. Accumulating evidence also demonstrated that increased circulating adiponectin levels in ACS were independently associated with a higher risk of recurrent cardiovascular events including death, myocardial infarction and heart failure [16]. Therefore, whether circulating chemerin levels in ACS are associated with a higher risk of recurrent cardiovascular events should be investigated in the future.

Although the levels of adiponectin in the complex lesion group and the simple lesion group were significantly lower than those of the control group in our previous study, no differences were observed between the complex and simple lesion groups [17]. In the present study, the Gensini score was used to assess the severity of coronary stenosis in CAD patients. Although significant correlations between the two adipokines and the Gensini score were found in patients with SAP, neither chemerin nor adiponectin was correlated with the Gensini coronary score in patients with ACS. Our colleagues Gao et al. showed no correlations between circulating chemerin or adiponectin and the Gensini coronary score in a previous study [44], while another study performed by Yan et al. found that chemerin levels were associated with the Gensini score even after adjusting for age, sex, and other established risk factors of CAD [31]. We speculate that enrolled patients with different clinical types of CAD may contribute to this discrepancy. Yan et al. may enroll majority patients with stable CAD in their study, while Gao et al. may enroll majority patients with ACS.

## Conclusions

In conclusion, our study presented for the first time novel data that reveal increased plasma chemerin levels in ACS but not in SAP. More importantly, we found that increased plasma chemerin levels were independently associated with the presence of ACS. Thus, circulating chemerin may serve as a novel biomarker of ACS but not of SAP. However, the present study has some limitations. There has been no follow-up with these ACS patients to assess the short- and long-term prognostic significance of chemerin levels, which should be investigated in the future. The relationship between chemerin and cardiac function also should be investigated in experimental study.

## Abbreviations

ACS: Acute coronary syndromes
ACEI: Angiotensin-converting enzyme inhibitor
AMI: Acute myocardial infarction
ARB: Angiotensin receptor blocker
CCB: Calcium channel blocker
CAD: Coronary artery disease
CRP: C-reactive protein
GLU: Fasting glucose
HDL-C: High-density lipoprotein cholesterol
LDL-C: Low-density lipoprotein cholesterol
LVEDD: Left ventricular end-diastolic dimension
LVEF: Left ventricular ejection fraction
SAP: Stable angina pectoris
TC: Total cholesterol
TG: Total triglycerides
UAP: Unstable angina pectoris
